# Turning on the we-mode: a systematic review on joint action principles for promoting collective pro-environmental engagement

**DOI:** 10.3389/fpsyg.2025.1642312

**Published:** 2025-10-15

**Authors:** Shahryar Sarabi, Marleen Gillebaart, Denise de Ridder

**Affiliations:** ^1^Department of Social, Health, and Organisational Psychology, Utrecht University, Utrecht, Netherlands; ^2^Information Systems in the Built Environment (ISBE) Group, Department of Built Environment, Eindhoven University of Technology, Eindhoven, Netherlands

**Keywords:** joint action, collective engagement, pro-environmental action, prosocial behavior, systematic review

## Abstract

Collective pro-environmental engagement of communities is vital for addressing climate change through system-wide transformations. To promote such engagement, individuals must go beyond their immediate personal interests, requiring activation and promotion of pro-social behaviors. In this review we aimed to explore joint action as a way to “boost” collective action approaches beyond specific frontrunner groups. In recent years, joint action (i.e., social interaction whereby individuals coordinate their actions to bring about a change in the environment.) has received significant attention as an approach that can bring about various pro-social behaviors. We conducted a systematic literature review to identify the pro-social outcomes associated with joint action and discuss its potential to promote collective pro-environmental engagement. Our analysis revealed two types of pro-social behaviors: those related to group functioning (togetherness, perspective taking, and cooperative behavior) and those tied to group performance (commitment, agency). These behaviors can be effectively promoted by joint action, as witnessed by medium to large effect sizes. We therefore argue that these findings offer a promising pathway for leveraging joint action as a means to enhance collective pro-environmental engagement across a broad segment of the population, and ultimately provide effective climate governance strategies.

## 1 Introduction

In the Anthropocene, society grapples with climate change as one of the most challenging problems of our time. Under the Paris Agreement, governments worldwide have committed to limit the global temperature rise below 2 °C and make an effort to limit it further to 1.5 °C, which requires reaching net-zero emissions by around 2050 (Fankhauser et al., 2021). Reaching these targets requires strong commitment from governments and substantial behavior change of a wide-range of other actors (Hampton and Whitmarsh, 2023). Despite the efforts made, incremental policy changes alone have proven insufficient to comprehensively respond to climate change (Tosun and Schoenefeld, 2017). It is therefore increasingly acknowledged that addressing climate change calls for system-wide transformations that should be approached as a collective action problem (IPCC, 2022).

Understanding climate change mitigation as a collective action issue stands in contrast with traditional approaches that focus on individuals. While successful to some extent (e.g., Abrahamse et al., 2005; Möser and Bamberg, 2008), individual approaches may fall short in mobilizing the united effort that is required for transformative change (Rees and Bamberg, 2014). Moreover, focusing solely on individual pro-environmental behavior can lead to feelings of helplessness and inaction of the individuals involved (Gunderson, 2023; Salomon et al., 2017). As a result, collective action—defined as action taken together by a group of people whose goal is to improve their condition and achieve a common objective (Wright et al., 1990)—has emerged as a potentially more effective approach for addressing climate change (Amel et al., 2017; Fritsche et al., 2018; Poteete et al., 2010).

In the realm of collective action research, competitive and cooperative approaches are distinguished (Wright, 2009). The competitive understanding of collective action typically emphasizes clear boundaries between ingroup and outgroup members and highlights perceived injustice as the main driver of acting together (e.g., Agostini and Van Zomeren, 2021; Jurstakova et al., 2023; Van Zomeren et al., 2008). In contrast, the cooperative view does not so much focus on ingroup-outgroup differences but rather highlights inclusivity and compassion toward outsiders (Bamberg et al., 2015). Thus far, our understanding of the mechanisms driving people’s decisions to engage in collective action is mainly rooted in the competitive approach (Wright, 2009). This also applies to collective action research in pro-environmental behavior (e.g., Bamberg et al., 2015; Fritsche et al., 2018; Van Zomeren et al., 2010). Empirical research has found support for with the notion that members of a group seeing themselves as a collective “we” rather than individual “I” are more inclined to engage in behaviors aligned with the group’s pro-environmental norms and goals (Bamberg et al., 2015; Barth et al., 2021; Fritsche et al., 2018; Masson et al., 2016; Vesely et al., 2021).

Whereas these insights are important, they primarily relate to groups who are able and willing to organize themselves. Unfortunately, the actions of this committed minority may ironically reinforce the majority’s adherence to prevailing practices (Bolderdijk and Jans, 2021). It is therefore urgent to move beyond the avantgarde role of the environmental elite who are ahead of the crowd in their call for change in the sustainability domain (Tropp et al., 2021). We therefore aim to investigate the potential of joint action as a way to boost a cooperative way of collective action. Providing a larger and more diverse group of people (including those who lag a bit behind) with the opportunity to participate by promoting self-organization in underprivileged communities is imperative for an inclusive and fair sustainability transition. There is, however, a lack of systematic understanding of how we can foster collective pro-environmental engagement beyond elite groups. To address this knowledge gap, it is important to consider approaches that highlight a cooperative understanding of collective action, such as joint action.

Insights from the cooperative approach are important for fostering a shared environmental identity, cultivating a deep-rooted trust, and instilling a sense of collective agency across diverse communities. Addressing these issues is crucial for enabling societies to rise to the urgent challenges of climate change and environmental sustainability. Here, we posit that the answer to these questions rests on our ability to translate psychological insights from the cooperative approach to collective action into actionable strategies that speak to large groups of people. Specifically, we argue that the key to promoting cooperative collective action lies in *joint action*, defined as “any form of social interaction whereby two or more individuals coordinate their actions in space and time to bring about a change in the environment” (Sebanz et al., 2006, p. 70). The joint action concept is underpinned by a deep intrinsic motivation for working together (Melis, 2013) to the extent that people prefer to perform a task together even when acting individually is more efficient (Curioni, 2022). We therefore propose that mapping the mechanisms in joint action can close the knowledge gap between the need for effective climate change mitigation strategies and inclusive collective action that allows people to contribute to this challenge.

In this review, our objective is to map the opportunities of joint action insights for promoting collective engagement with climate change mitigation policies. To do so, we performed a systematic review^1^ on joint action research. First, we will map the critical mechanisms of joint action (working together on a common task) and how they contribute to a variety of prosocial behaviors that may in turn be instrumental to pro-environmental action. Based on this analysis, we will subsequently discuss potential pathways through which joint action insights can support collective pro-environmental engagement and how they can be implemented into policy arrangements.

## 2 Method

**Figure 1:**
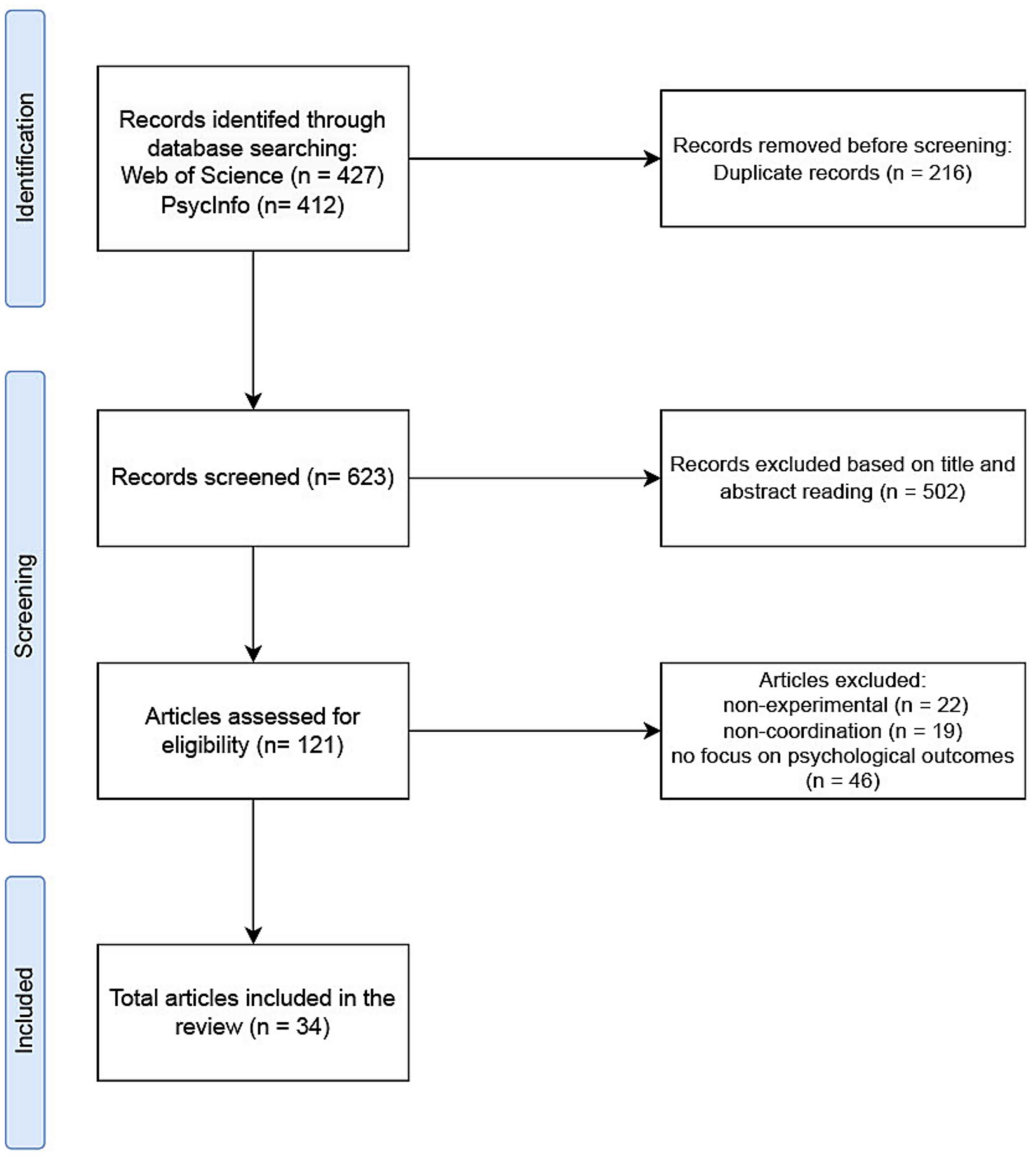
PRISMA flow diagram.

We mapped the behavioral outcomes of coordinated joint action by performing a comprehensive systematic literature review. Our review adheres to the Preferred Reporting Items for Systematic Reviews and Meta-Analyses (PRISMA) guidelines (Page et al., 2021), distinguishing between identification, screening, and inclusion of relevant studies, as illustrated in Figure 1.

### 2.1 Inclusion and exclusion criteria

Our inclusion criteria comprised (1) empirical studies that examined (2) the behavioral outcomes of coordinated joint action, and (3) were published in English. Moreover, we only included research published in peer-reviewed journals while excluding reviews, conference papers, books, book chapters, and gray literature. As a result, papers that focused primarily on the precursors of joint action were excluded. Moreover, as the emphasis of our study lies on uncovering joint action insights with potential applicability in sustainable engagement, we excluded studies exclusively and explicitly investigating synchronized coordinated action in highly protocolled lab tasks. Whereas synchrony emphasizes temporal alignment, joint action goes beyond that as it involves individuals interacting and planning their actions to achieve a common goal (Wallot et al., 2016). Finally, we did not consider studies focusing on clinical populations or non-human subjects.

### 2.2 Search strategy

For a thorough examination of potentially relevant research, we employed the databases of Web of Science (WoS) and PsycInfo. Our objective was to find papers addressing joint action. According to the definition of joint action proposed by Sebanz et al. (2006), “coordination” describes the interaction between people involved in a joint action. Therefore, to focus the review on joint action that bring about a change in the environment we included coordination (and terms with the same root) in the search terms. Moreover, after a general scan of the relevant literature we found that terms “cooperation” and “collaboration” are also frequently used to refer to similar types of joint action, therefore we included them in the search terms for a move comprehensive overview of the literature. Consequently, the search terms we used were: ((coordinat* OR cooperat* OR collaborat*) AND “joint action”) to search the title, keywords and abstract fields of the indexed literature. To ensure the quality of the publications, we selected those indexed in either the Social Sciences Citation Index (SSCI) or the Science Citation Index Expanded (SCI-EXPANDED). The search was carried out on November 21, 2023. The search in WoS resulted in 1025 publications. Upon initial examination of the retrieved publications, their diversity across various domains was evident. To refine our focus specifically on joint action research from a psychological perspective, while still conducting a comprehensive scan, we applied the WoS Citation Topics Meso filter “Neuroscanning” which covers studies focusing on behavioral mechanisms (Clarivate, 2021). This allowed us to exclude studies focused on subjects such as Robotics, Gait & Posture, Management and Political Science. This strategy resulted in 427 publications from WoS. A parallel search on PsycInfo with the same terms yielded 412 publications. In total, our searches identified 839 publications. After removing 216 duplicates, we included a collection of 623 unique publications for review.

### 2.3 Screening

Screening of selected publications was conducted in two stages. First, we processed the publications into ASReview software (ASReview LAB developers, 2024) to analyse their titles and abstracts. ASReview uses active learning techniques to train a machine learning model which predicts relevant text from a limited number of labeled examples (Van de Schoot et al., 2021). By accelerating the screening of titles and abstracts, this open-source software enables researchers to have an overview of the most relevant studies efficiently. Following this initial screening, we identified 121 publications that met our earlier specified criteria for a more detailed analysis. We then extracted and retrieved the full texts of these selected publications. To reduce the risk of bias, we selected only experimental studies that provided detailed explanations of the recruitment process and experimental protocol. Moreover, screening was conducted collaboratively, with three researchers involved in the process. After a comprehensive review of the full texts, we included 34 publications in our review. Of the 34 selected papers, 29 were published after 2013. Lastly, we looked at publications considered in other review studies on joint action including Loehr (2022) and Sebanz and Knoblich (2021) to ensure the inclusion of studies focusing on the behavioral outcomes of joint action.

## 3 Context building: the potential of joint action for encouraging pro-environmental engagement

**Figure 2:**
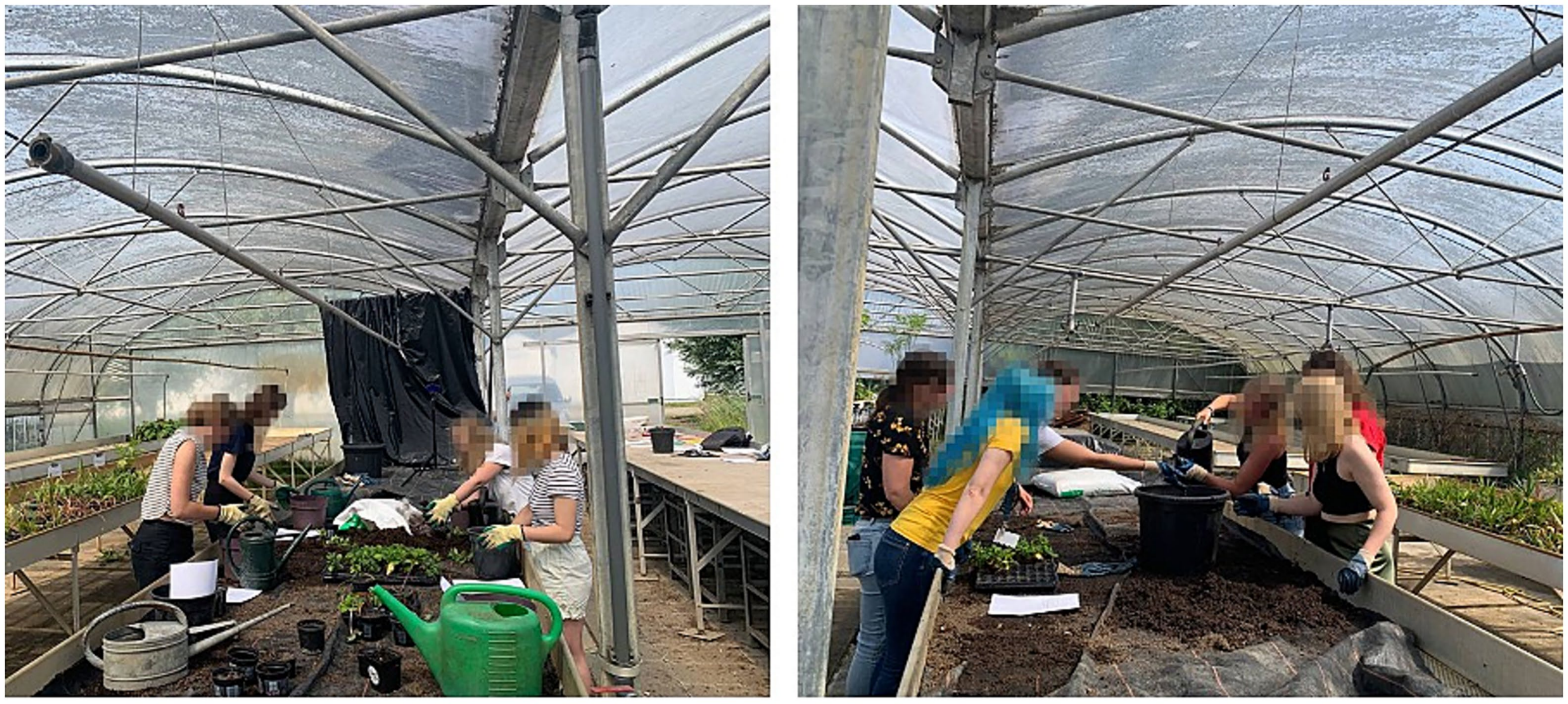
Joint action plant potting experiment—left image: individual condition, right image: joint condition.

Collective action is considered a vitally important avenue for addressing societal challenges, including the energy transition (Amel et al., 2017; de Ridder et al., 2023). Over the past decades, collective action research has documented the beneficial outcomes of citizens working collaboratively on the provision of energy, food and other goods and services by mapping the implicit and explicit institutional statements (strategies, norms, rules, sanctions) that are responsible for these successes (McGinnis, 2011). However, in doing so these studies have paid little attention to the grounding of these statements in actual psychological processes, precluding our insight in how we can encourage collective action and what kind of mechanisms are responsible for people getting together and cooperate on a common objective. The concept of joint action—i.e., working together on a common task by coordinating one’s actions (Sebanz et al., 2006)—has the potential to address this knowledge gap. Critically, a joint action approach posits that people do not need to have a common objective beforehand but that a shared goal emerges from working together (Sebanz and Knoblich, 2021), for example when they synchronize their steps when walking together (Atherton et al., 2019) or coordinate their actions to remove a pile of sand (Michael et al., 2016). Why is it that people would be willing and able to engage in joint action? Research into the brain’s mirroring properties suggests that people can have direct first-person access to the feelings, thoughts, and intentions of others (Rizzolatti and Sinigaglia, 2016). These basic mechanisms of resonance and simulation allow people to prepare for joint action by forming representations of each other’s actions and the relation between them. This enables them to predict each other’s upcoming actions, which, in turn, facilitates coordination (Sebanz and Knoblich, 2021). Joint action has been shown to enhance trust and social bonding (Carr and Walton, 2014), collective agency (Loehr, 2022) and commitment (Michael et al., 2016), promote cooperation in social dilemmas (Wiltermuth and Heath, 2009) and, more generally, promote prosocial behavior (Tomasello and Vaish, 2013). Despite these known beneficial effects, research on the effects of joint action in naturalistic group settings such as communities or neighborhoods is scarce. Initial research has demonstrated that joint action also generates beneficial effects outside the lab in a series of field experiments employing a plant potting paradigm where people work together in either low (i.e., a group of people individually pots a plant) or high coordination (i.e., a group of people collectively pots a plant), including people from underprivileged neighborhoods. Compared to low coordination, people coordinating their actions with other group members reported greater connectedness with the group, greater collective agency, and increased engagement with the task (see the data from our ongoing experiments https://osf.io/8qtge/?view_only=1cf712cc9237454ead83a7c9dfbc04ae, as well as an example of the experimental set-up used in Figure 2).

## 4 Results

### 4.1 Overview of the selected publications

**Figure 3:**
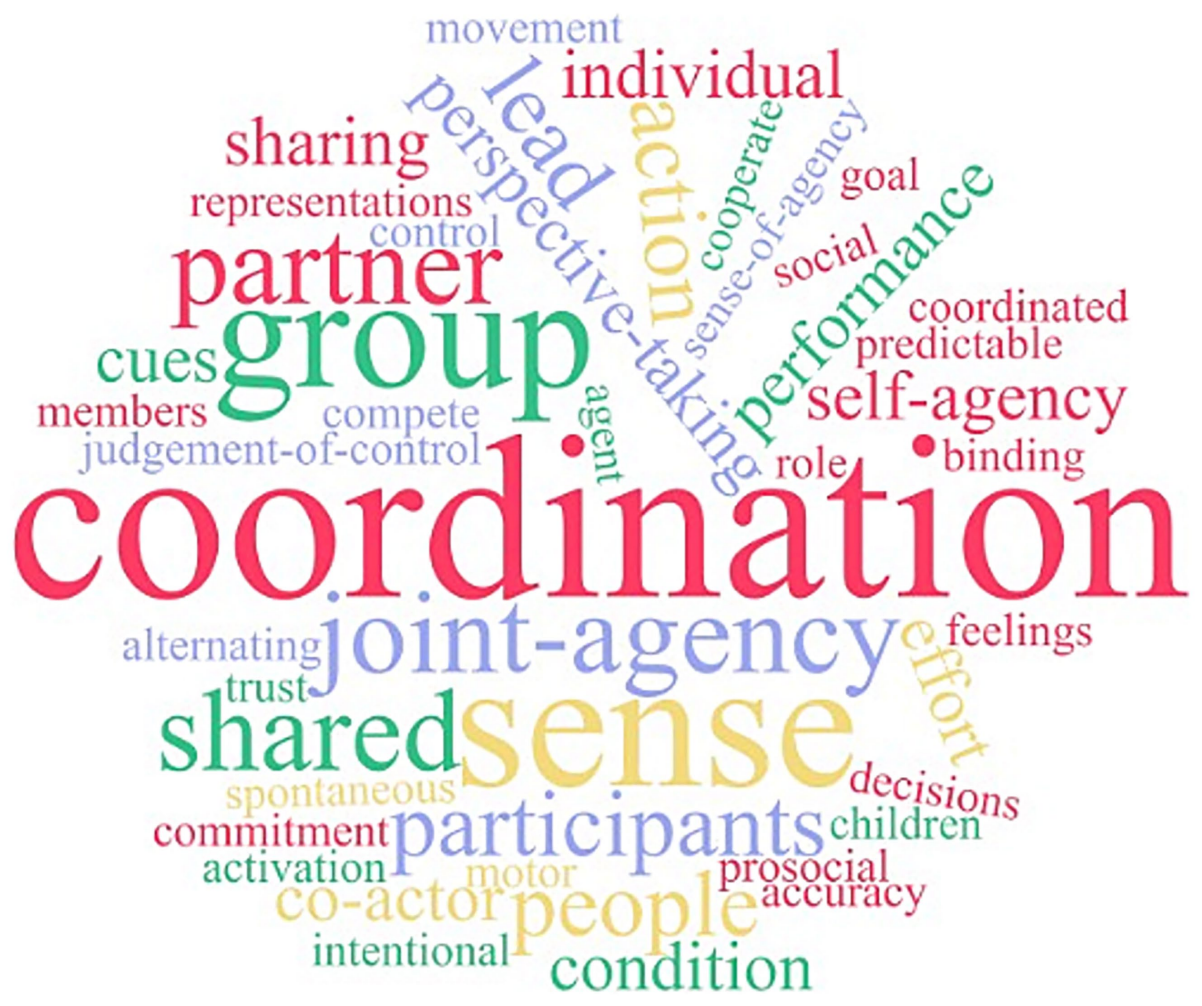
Word cloud of the main findings of the reviewed papers.

From this overview it is evident that most studies employed lab-based coordination tasks. Moreover, the majority of studies examined pairs of participants rather than groups consisting of at least three people; groups larger than four were absent from our overview. Typical coordination tasks include joint tone production (musical instruments or computer-based key-tone exercises), object movement control (physical or virtual using a joystick), joint movement, or block arrangements (e.g., Lego model making) in which pairs of participants engaged in an interactive task to achieve a joint goal. All studies comprised lab-based tasks and no studies were performed in a field setting. The word cloud presented in Figure 3 provides an overview of the main findings of the reviewed papers. Supplementary Table S1 presents an overview of the publications selected for review with details about the joint action task, conditions, sample size, number of participants, outcomes as well as the main findings of each record relevant to this study and the associated effect size. As shown in Supplementary Table S1, insofar effect sizes were reported, they varied from medium (0.02 < ŋ^2^ < 0.06 or Cohen’s d = 0.2) to large (ŋ^2^ > 0.14) (Cohen, 1988).

### 4.2 Behavioral outcomes of joint action

**Figure 4:**
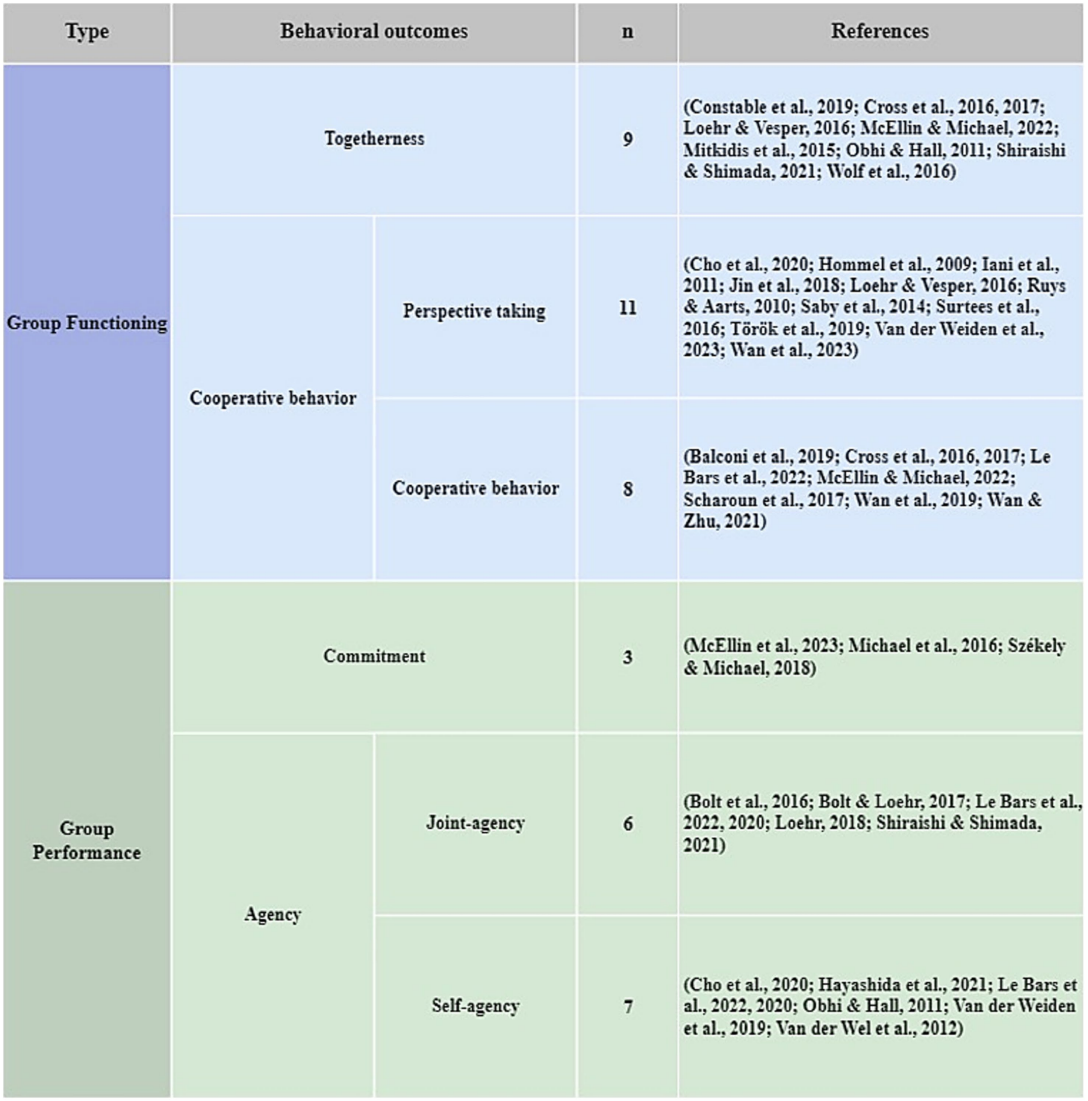
Behavioral outcomes of joint action.

A variety of outcomes of engaging in joint action tasks were discussed in the reviewed publications, which we classified into four comprehensive categories, with inspiration from prior categorizations (Fernández Castro and Pacherie, 2021; Michael et al., 2020): (1) Togetherness, (2) Agency, (3) Commitment, and (4) Cooperative behavior. Togetherness relates to considering oneself as being part of a group and comprises three core elements: a sense of shared identity, cohesion, and trust. Sense of agency is a second focal outcome in joint action research and generally defined as having a sense of voluntary control over one’s actions and their effects (Zapparoli et al., 2022). For the purpose of the present review, we are particularly interested in collective sense of agency referring to control over actions as a group. The third outcome of interest concerns a sense of commitment to the task the group is engaged in. Task commitment is distinguished from other joint action outcomes as it may be driven by the expectation of reciprocity from other partners independently from other mechanisms (Michael et al., 2020). The fourth outcome relates if partners show cooperative behavior by making prudent decisions to maintain the coordination (McEllin and Michael, 2022) or whether partners working together on a task take each other’s perspective in their actions (Wan et al., 2023). Most studies do not examine one single outcome but a variety of outcomes (which they do not explicitly relate to each other). Figure 4 lists the studies according to these four types of outcomes. Some of the studies are assigned to more than one category.

#### 4.2.1 Togetherness

Nine studies discussed the potential impact of joint action on people’s experience of being part of a group and their perception toward other people in the same group, including a sense of cohesion and closeness, and trust. Two studies demonstrate that participants who are required to engage with each other actively to accomplish a task (i.e., a producing a joint tone), may experience a greater sense of ‘we-identity’ irrespective of whether they were the initiator or the responder of behavioral coordination (Obhi and Hall, 2011; Shiraishi and Shimada, 2021). Another study showed that just considering oneself as a group member proved sufficient for prioritizing the processing of information relevant to one’s group independent of knowing other partners or sharing preferences with them (Constable et al., 2019). Several other studies provide evidence that joint action can enhance group cohesion and sense of closeness. For example, cuing pairs of participants to attend to the same part of a screen was sufficient to enhance a sense of social bonding as compared to asking them to look at different parts of a screen (Wolf et al., 2016). These effects are also present beyond pairs to the extent that groups of three or four people imagining joint action (rather than actually working together) reported a greater sense of cohesion with group members, possibly because they think of themselves in less individualized ways (Cross et al., 2016, 2017). Other studies show that joint action can enhance a sense of trust between partners. In one study using a trust game paradigm, it was found that participants who receive signals from their partners (regardless of whether these signals were useful or redundant) report greater trust as compared to when they do not interact with their partners (McEllin and Michael, 2022). Similar findings were reported in a study that required pairs of participants to build car models with LEGO bricks in four sessions, while their heart rate was being measured. In one group, each session was followed by a public goods game while in the other group there was no public goods game. This study found that in the public goods game condition participants trusted each other more, which was marked by higher heart rate synchrony, and that a higher heart rate synchrony predicts higher expectation (preferences or believe about the behavior of others) of return in the economic game. Accordingly, they argued that a partner performed a task which involves the risk of trust only if they expected that their effort would be reciprocated (Mitkidis et al., 2015). Both studies insinuate that building expectations of reciprocity, based on previous interactions, caused partners to exercise trust and cultivate a reputation as trustworthy partners. Engaging in joint action may also cause people to form representations of shared goals, according to a study that showed that having a shared goal (preparing a duet) result in less performance errors compare with an individual goal in a musical transfer or learning task (Loehr and Vesper, 2016). Further research on joint action within different contexts and within groups of more than two participants may further elucidate the potential benefits of shared goals on the experience of togetherness. Taken together, these studies show that joint action can lead to a heightened sense of cohesion, togetherness and trust especially when people expect their partners to reciprocate with cooperative behavior.

#### 4.2.2 Cooperative behavior

We identified 19 studies delving into the question of how joint action may encourage perspective-taking and cooperative behavior. Of these, eight papers explored in what way joint action may enhance cooperative behavior with a focus on how reciprocity supports cooperative behavior (Le Bars et al., 2022; McEllin and Michael, 2022; Scharoun et al., 2017). A pegboard task experiment whereby a participant-confederate pair worked together to move a peg from one side of the board to the other side revealed that in the condition where a helpful confederate was present (as compared with the condition with an unhelpful confederate), the peg was moved further so as to reduce the effort required by the confederate (Scharoun et al., 2017). This suggests that participants attempted to reciprocate perceived cooperation. Another study employing a dictator game paradigm found that partners tended to donate more when they made an effort to interact and did send useful signals to one another, suggesting that they may have had reciprocal motives (McEllin and Michael, 2022). The role of reciprocity as a potential mechanism underlying the consideration of others is also evident from studies showing that people perform better in a cooperative task when they first exchange a gift (Balconi et al., 2019) or when they have equal (symmetric) roles in performing a task (Le Bars et al., 2022).

Interestingly, the impact of joint action on cooperative behavior is also evident in children (Wan et al., 2019; Wan and Zhu, 2021). In two studies examining pairs of children engaging in a musical task, it was found that coordinated joint action, in which pairs are continuously engaged with each other, cooperative behavior (e.g., sharing or donation of objects) increases beyond just having a shared goal (Wan et al., 2019), especially insofar fine-grained coordination is involved (Wan and Zhu, 2021).

Another key outcome of joint action is perspective-taking and partner co-representation. We found 11 studies providing that partners in joint action take each other into account when planning their actions. Partners may forgo action efficiency to accommodate each other’s perspectives, as shown in a study finding where participants sacrificed the efficiency of their own action when it reduced their partner’s effort in an attempt to maximize the efficiency of their combined effort (Török et al., 2019). Similar findings were reported in a study showing that young children incorporate the role of a partner (i.e., an adult experimenter) into their action plan when performing a joint Simon task (Saby et al., 2014). In an experiment in which participants coordinated to learn and perform a piano melody, Loehr and Vesper (2016) found that partners made more errors in the individual goal conditions than in the shared goal conditions, suggesting that they developed a shared representation of their goal. However, having a shared goal may not be required for perspective-taking as people may also spontaneously adopt the perspective of their partner in joint action (Surtees et al., 2016). Overall, perspective-taking appears to be more prevalent in interdependent joint action (Cho et al., 2020; Hommel et al., 2009; Iani et al., 2011; Jin et al., 2018; Wan et al., 2023).

In two Joint Simon task experiments, Iani et al. (2011) found that shared representation will be activated only when partners cooperate but not when they compete. This is consistent with the findings of Hommel et al. (2009), who also found a significant effect of spatial correspondence in cooperative groups but not in competitive groups, indicating the emergence of interactive Simon effect when partners cooperate but not when they compete. These are aligned with Jin et al. (2018), who found that in a joint action game in which a child and an adult acted in competitive or cooperative conditions, coordination enhances the children’s performance in understanding other’s desires and perspective-taking. Cho et al. (2020), also investigating EGG signals from participants in a competitive or cooperative visuomotor joint action experiment, found evidence for brain activity supporting action co-representation in cooperative joint actions.

However, Ruys and Aarts (2010), in two joint Simon task experiments, found that shared representation can also emerge when co-actors compete due to attending to a co-actor’s intentions. Another factor that can affect action co-representation is the predictability of a partner. Van der Weiden et al. (2023), in a two-stage experiment including an induction phase followed by a joint Simon task, found that the predictability of a partner’s actions can modulate self-other integration, with self-other integration being stronger for predictable than unpredictable partners.

#### 4.2.3 Commitment

Three studies provided evidence that joint action may lead to an increased sense of commitment to a shared task (McEllin et al., 2023; Michael et al., 2016; Székely and Michael, 2018), shedding more light on the conditions under which joint action can boost a sense of commitment. A seminal study by Michael et al. (2016) describes a series of experiments where participants watched videos featuring two individuals engaged in a joint task under two distinct scenarios: high-coordination and low-coordination. The study revealed that observers tend to perceive individuals in the high-coordination scenario as more likely to resist external temptations and stay committed to the task. They also found that taking the perspective of the helper enhances observers’ perception of commitment, which supports the hypothesis that coordination enhances commitment by creating a sense of social obligation for partners. Two other studies found that participants have higher sense of commitment when coordinating with partners who tailor their behavior to ensure successful and smooth coordination than non-adaptive partners (McEllin et al., 2023; Székely and Michael, 2018). Together, these findings suggest that a joint action task requiring partners to make an effort to adjust their actions to those of their partners create a sense of debt toward each other, which in turn motivates them to remain committed to their partner(s) and to the task.

#### 4.2.4 Agency

Eleven studies examined the link between joint action and a sense of agency, either self-agency, joint agency or both. A common understanding of these studies is that sense of agency increases when partners perceive the outcomes of the action to be the result of their own efforts. Generally speaking, a higher sense of self-agency is observed for self-produced actions than for other-produced actions (Van der Weiden et al., 2019). In a skill learning task with two individual and dyad condition, Van der Wel et al. (2012) found that the sense of self-agency significantly increases when moving from joint action to individual action, showing that the increase in sense of self-agency might be related to the learning context of the task. The sense of self-agency is higher in particular when partners are focused on their own given target in competition with others (Cho et al., 2020). This aligns with studies showing a stronger sense of self-agency among initiators of a joint action as compared to followers, likely due to an increased sense of outcome responsibility (Bolt et al., 2016; Le Bars et al., 2020, 2022; Obhi and Hall, 2011). Other research suggests that joint action can enhance self-agency when partners align on a shared goal and feel accountable for their own roles. For example, it has been shown that goal sharing by means of cooperative joint action can significantly improve sense of self-agency on a pre-reflective level (intentional binding) as compared with independently working on a task (Hayashida et al., 2021).

There is consensus among studies that engaging in joint action may create a sense of joint agency. One of the main factors affecting such an experience lies in being able to accurately predict the actions of (the) partner(s) and the outcome of joint action (Bolt et al., 2016; Bolt and Loehr, 2017; Loehr, 2018; Shiraishi and Shimada, 2021). In a series of experiments in which two partners produced tones in alternation while receiving explicit or implicit feedback regarding their performance, it was found that participants derive their feelings of joint agency (i.e., shared control and shared responsibility) from the success of their group as a whole (Loehr, 2018). Furthermore, when the predictability of a partner’s actions was manipulated, it was found that people report a higher sense of joint agency when working with more predictable partners (Bolt and Loehr, 2017). Similar experiments revealed that participants producing tones in alternation (as compared with sequential) were more successful in coordinating their actions, resulting in reports of higher joint agency, regardless they were initiators or followers of the task (Bolt et al., 2016; Shiraishi and Shimada, 2021). Likewise, it has been reported that partners with asymmetric roles and/or gains from a joint task experience a lower sense of joint agency, possibly due to the inability to co-represent each other’s intentions (Le Bars et al., 2022; Le Bars et al., 2020). Taken together, these findings suggest that symmetrical coordination enhances the feeling of joint agency among partners.

## 5 Discussion

**Figure 5:**
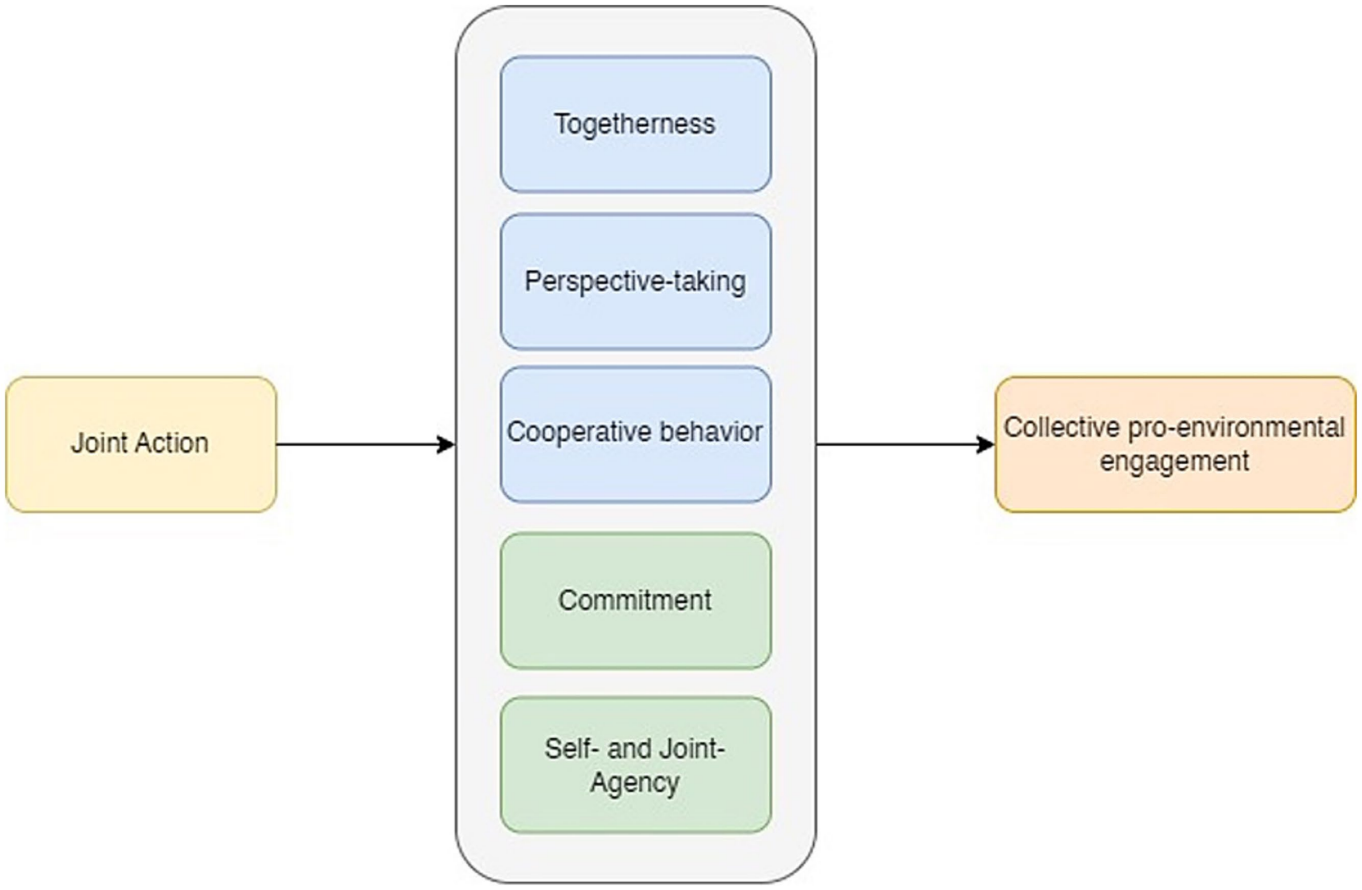
Psychological outcomes of joint action fostering collective pro-environmental engagement.

Previous research on competitive collective action has investigated in what way belonging to a group may foster cooperative effort by emphasizing that people need an outgroup to get into action (Fritsche and Masson, 2021; Van Zomeren et al., 2010). Our review of fundamental psychological research on joint action complements that approach with knowledge highlighting the benefits of cooperative collective action to boost collective pro-environmental engagement beyond elite groups. The joint action concept is largely unknown outside the psychological community but lends itself well to appreciate the dynamics of working together in coordination on a common cause. It builds on the notion that people are uniquely able and motivated to collaborate and experience inherent pleasure from collaboration. Our review clearly attests to the beneficial effects of engaging in activities that are guided by joint action principles. We documented significant positive outcomes that we grouped into the five comprehensive categories of togetherness, agency, commitment, and perspective taking and cooperation to organize the wide variety of prosocial behaviors that have been reported in the literature (see Figure 5). Overall, effects were medium to large sized, which is impressive and much larger than effects generally reported in the psychological literature (e.g., Lipsey and Wilson, 1993; Richard et al., 2003). Whereas alternative categorizations of prosocial outcomes have been discussed (e.g., Dunfield, 2014), our classification of outcomes reveals beneficial effects of join action at two levels relevant for collective action, group functioning (togetherness, perspective taking, and cooperative behavior) and group performance (commitment, agency). Our review provides substantial evidence for the impact on both components as, for example, illustrated by the effects of joint watching of a screen on social bonding (Wolf et al., 2016) and of gift exchange on subsequent performance in a dictator game (Balconi et al., 2019). Although it is well known that group functioning (e.g., feelings of togetherness and working together) is important for accomplishing a collective task, so far it has not been well understood how to create optimal conditions that foster group functioning and group performance to the extent that people feel good and do well.

We argue that joint action insights from psychological science are relevant for understanding the role of citizen engagement with sustainability challenges as they may support the design of collective action arrangements. As of now, and in spite of the increasing popularity of citizen collectives as an instrument to govern the sustainability transition, basic knowledge on joint action is absent from attempts to promote smooth collaboration within these collectives. Yet, it is known that the magic of collaboration does not happen automatically when people are simply put together in an energy community (Blasch et al., 2021) and that attempts to get people working together may even backfire when they feel that their contribution is taken for granted (Bal et al., 2021). It is therefore urgent that micro-level insights into the group dynamics of coordination that have been discovered in lab settings are linked to a macro-level approach that allows for the examination of joint action in real world conditions. All in all, then, behavioral notions related to coordination and collaboration mechanisms within diverse groups are essential for understanding when and why people will have a strong experience of joint agency that in turn may create strong commitment to the sustainability transition. As such, we claim that joint action can close the knowledge gap between the need for effective climate change mitigation policies and inclusive collective pro-environmental engagement. Employing these insights to govern the transition by providing and/or stimulating arrangements for joint action, especially for people who do not gather naturally to engage in collective action, has the potential to make significant steps toward a sustainable society.

### 5.1 Recommendations for policy making

While the importance of collective action has been recognized in previous studies (Amel et al., 2017), so far it has been unclear how policies could “turn on the we-mode” by facilitating people to operate as a group, especially insofar working in coordination is concerned. We believe that implementing joint action principles into existing interventions for pro-environmental action or into the design of new ones is a promising avenue for encouraging collective engagement with pro-environmental action. Current climate policies are already focused on involving larger groups of people by emphasizing the importance of collective efficacy (Bandura, 2000; Fritsche and Masson, 2021), social norms (Goldberg et al., 2020), dynamic norms (Sparkman and Walton, 2017), and tipping points (Nyborg et al., 2016) as important avenues for increasing citizen engagement with sustainability issues. Although these approaches are compelling, they are mute about *how* to improve collective efficacy, change norms, or create tipping points so as to build greater community engagement. We propose that inserting joint action elements into interventions could fill this gap.

To successfully implement climate policies, it is important to actively support community involvement by good governance arrangements. Interestingly, the seminal work of Ostrom (1998) on collective action governance has noteworthy parallels with elements that we identified in fundamental joint action research, including reputation, trust, and reciprocity as crucial ingredients for collective engagement. Ostrom argues that the mutually reinforcing relationships between the trust that an individual has in others, the investment people make in trustworthy relationships and the probability that individuals will use reciprocity, are the core relationships affecting people’s cooperative behavior in collective actions. Our review shows that joint action actually enables people to build expectations of reciprocity, signal the willingness to accommodate each other’s expectations, and to manage one’s reputation as a trustworthy partner. Therefore, joint action can be considered as a potential instrument for reinforcing the underlying behavioral mechanisms necessary for promoting cooperative behavior in collective climate actions. Joint action can ignite a sense of commitment among members of a community by providing settings in which people can experience that partners will act cooperatively. This also aligns well with models of earth system governance (Biermann et al., 2010) and collaborative governance (Ansell and Gash, 2008; cf. De Dreu et al., 2023; Mayer, 2014), positing that direct communication in a collaborative context allows for experiencing closeness, cultivating trust, building commitment, and a shared perspective in such a way that these outcomes may mutually reinforce each other (Fernández-Castro and Pacherie, 2023).

In practice, joint action can be considered an instrument for promoting cooperation among people when organizing collective climate actions. For example, to establish energy communities as an innovative approach to support clean energy transition (European Commission, 2025), people need to cooperate closely and join forces. In this process, joint action can play a key role. By embedding joint actions within the organizational process of energy communities, it is possible to promote the underlying mechanisms for cooperative behavior among people, thereby enhancing citizen participation in energy transition.

### 5.2 Recommendations for future research

In considering the ample opportunities for future research on the role joint action in collective climate action, some limitations of the present research should be mentioned. First, many of the reviewed studies provide evidence that joint action generates prosocial outcomes in controlled lab settings. Among the reviewed studies, there are no field-based approaches, although that could be due to the limitations in the search strategy of this study. However, adopting a field-based approach is necessary to further establish robustness and effectiveness of joint action mechanisms for real-life behavioral outcomes. Moreover, most joint action manipulations relate to tasks that do not lend themselves to be employed directly in real life interventions. Whereas, for example, joint tone production (e.g., Obhi and Hall, 2011) or the Joint Simon task (e.g., Iani et al., 2011) are valid protocols to generate joint action in the lab, it is hard to imagine how they could easily be used outside this controlled environment. However, other studies have employed tasks that lend themselves better for translation into interventions, especially insofar it relates to manipulations of synchronized walking (Atherton et al., 2019) or playing music together (Loehr and Vesper, 2016). Focusing on translating these mechanisms to more ecologically valid settings in field studies is a promising avenue for further research. Moreover, synchronous actions and joint actions appear to be partially overlapping concepts, with synchrony focusing on time alignment and joint action emphasizing planning toward a common goal. Despite the marked differences between joint action and synchrony, it should be noted that beneficial prosocial outcomes are quite similar. Another limitation lies in lack of studies examining generalization potential. Right now, it is not known to what extent the effects of participating in joint action generalize to related tasks that are not directly addressed in the experimental manipulation. Future research should examine whether these positive effects last longer in time beyond immediate task performance and whether they pertain other (related) tasks as well. It is also not known whether effects generalize to (affiliated) groups of people that were not involved in the primary task. Yet, it has been reported that even after only 2 min of synchronous walking, non-Roma Hungarians reported more liking of a Roma partner and more empathy toward the Roma as a group, suggesting that positive effects may transfer beyond the partners who were directly involved in the task (Atherton et al., 2019). More insight into effects of group composition (e.g., homogeneous or heterogeneous groups) is critical for examining the implementation potential of joint action principles into pro-environmental policies. A similar caveat should be made regarding group size as the majority of reviewed joint action studies employed pairs of participants rather than groups. Future research should examine the effects of joint action in larger groups. Still, as of now, it seems that joint action can be used to involve broader groups of people who do not spontaneously get together because of a common interest in sustainability matters. Interestingly, it has been shown that many people who are now active in the environment were attracted by the opportunity of doing some together with other people in their community rather than doing something for the environment per se (Sloot et al., 2019). Joint action may thus be employed to engage these groups by speaking to their motivation for getting together and enjoy doing something together (Curioni et al., 2022).

Although we need to know more about these issues, for now we conclude that joint action insights provide a powerful route for encouraging engagement with collective pro-environmental action in real world settings. After all, deep engagement with pro-environmental action requires a mindset that motivates people to go beyond their immediate personal interests and make efforts for the good of society-at-large (de Ridder et al., 2023; Goldberg et al., 2020). Considering the current recognition of people’s motivation to engage in pro-environmental behaviors, our findings regarding the effects of joint action arrangements provide novel pathways to make this a collaborative effort that speaks to large parts of the population and lends itself for implementation in sustainability policies.

## Data Availability

The original contributions presented in the study are included in the article/Supplementary material, further inquiries can be directed to the corresponding author.

## Footnotes

^1^Considering the heterogeneity of the outcomes of joint action we were unable to perform a meta-analysis.
