# Supplementary material for: Turning on the we-mode: a systematic review on joint action principles for promoting collective pro-environmental engagement
Source: Front Psychol. 2025 Oct 15;16:1642312. doi: 10.3389/fpsyg.2025.1642312 (PMC12568691; doi:10.3389/fpsyg.2025.1642312)
Supplement: Supplementary file 1 [file Table_1.DOCX]

Supplementary Material

**Overview of the reviewed publications**

Table S1- Overview of the reviewed publications (in alphabetical order)

| Reference | Task | | Conditions | Sample size | | Group size | Behavioral measures | Main findings | Effect size |
| --- | --- | --- | --- | --- | --- | --- | --- | --- | --- |
| (Balconi et al., 2019) | Joint social task comprising a gift exchange during a general cooperative task | | (Condition: baseline, pre- Vs. post-gift exchange) AND (Role: donor Vs. receiver) AND (Gift: material Vs. experiential) | 32 female participants (Mage = 22.59) | | 2 | Accuracy; Reaction time | A behavioral performance increase was observed after gift exchange, with accuracy improvement and response times decrease. | Significant effect for Condition (before vs. after gift exchange) on performance (accuracy: η2 = .34, reaction time: η2 = .39). |
| (Bolt & Loehr, 2017) | Sequence tone production | | High-predictability Vs. low-predictability partner | 48 participants (17 male, Mage = 19.69) | | 2 | Mean agency rating | People reported stronger feelings of shared (as opposed to independent) control when they coordinated with the more predictable partner. | significant effect of partner predictability on judgement of the agency (d = .46). |
| (Bolt et al., 2016) | Sequence tone production | | (Alternation Vs. Sequential ) AND (leader Vs. follower) | 48 participants (12 male, Mage= 21.10) | | 2 | Mean agency rating; mean coordination score | Symmetric coordination causes a higher sense of joint agency than asymmetrical; coordination is more successful in alternating. | Significant effect of task on the sense of agency (Exp1: d ( Alt vs. Seq for follower) = .5, d ( Alt vs. Seq for leader) = .77, Exp2: d (Distant-Alt vs. Seq)= .39, d(I-Alt vs. Seq) = .48); Significant effect of task on coordination score (d (Immediate-Alt vs. Seq) = 1.66). |
| (Cho et al., 2020) | Jointly control a circular cursor with a joysticks to reach a target | | Cooperative Vs. Competitive | 40 participants (20 female, Mage = 22.5) | | 2 (and one confederate) | Degree of control (agency measure); EEG data recording | higher sense of self-agency in competition; Cooperative and competitive conditions shape affect processes important for co-representation. | Significant effect of action type (cooperate Vs. compete) on the sense of agency (η2 = .568). |
| (Constable et al., 2019) | shape-label matching task | | Trial type (Match Vs. Mismatch) AND Identity (Self/Coactor/Stranger/Team/Stranger Team) | Exp1: 28 participants (12 male, Mage = 24.82); Exp2: 28 participants (6 male, Mage = 22.50), Exp3: 28 participants (8 male, Mage = 22.68) Exp4: 40 (14 male, 26 female, Mage = 25.43) | | 2 | Response time | Participants were faster to report whether a label and a shape were matching when they referred to a group they belonged to (we) than when they referred to a group they did not belong to; neither knowing individual group members of one's own group nor sharing preferences with these members was a necessary precondition. | Significant effect of we-prioritization on response time compared to ‘they’(Exp1: p = .003, dav = .48, Exp3: p = .048, dav = .30, Exp4: p=.02, dav =.27). |
| (Cross et al., 2016) | Coordinated rhythmic movement task followed by an economic game (including a public goods game and an investment game) | In-phase Vs. anti-phase (coordination) (and a control condition with no coordination). | | Exp1: 66 participants (19 males and 47 females, Mage = 19.17); Exp2: 44 participants (8 males and 36 females, Mage = 19.86); Exp3: 22 participants (4 males and 18 females, Mage = 18.73) | 2 | | Mean vector length (coordination measure); Mean public account donation (cooperation measure) | Coordination in a social context leads to cooperation not just in-phase synchrony. | Significant Effect of Movement Type (In-phase, Coordinated and Control) on cooperation score (η2 = 0.15). |
| (Cross et al., 2017) | mentally simulated Coordinated movement task followed by a public goods game | | (Walking alone Vs. walking in-step with others) AND (with both instructions and video Vs. only instructions). | Exp1: 88 participants (14 males and 74 females, Mage = 19.71); Exp2: 356 participants (177 males and 179 females, Mage= 19.57) | | 3-4 | Mean public account donation, mean cohesion change, Mean de-individuation | Mentally simulated coordinated movement tasks can increases group cohesion and thinking of oneself as a group member. | Significant Effect of Imagined Movement on cohesion (Exp1: η2= 0.11, Exp2: η2= 0.09); A main effect of Imagined Movement on de-individuation (Exp2: η2= 0.02). |
| (Hayashida et al., 2021) | Motor performance task (stop a moving object on a monitor by pressing a key when object reaches the center of a target) | | (Cooperative Vs. Independent groups) AND (observed action Vs. self generated) | Cooperative group: 26 participants (13 same gender pairs, 18 females, Mage=20.8), Independent group: 26 participants (13 pairs, 20 females, Mage = 20.2) | | 2 | Intentional binding (self-agency measure) | Goal sharing improved motor accuracy and strongly enhanced the intentional binding effects on both self-generated and observed actions compared to non-goal sharing condition. | Significant effect of group type (cooperative or independent) on intentional binding (ηp2 = 0.106). |
| (Hommel et al., 2009) | (Joint) Simon task | | (cooperative condition Vs. competitive) AND (spatial correspondence Vs. spatial non-correspondence) | 28 participants (25 females, 3 males; Mage = 20.6) | | 2 | Reaction time | The interactive Simon effect was restricted to the participants confronted with a likeable co-actor who was thought to create a positive relationship. | Significant effect of correspondence in the positive-confederate group (ηp2 = .57). |
| (Iani et al., 2011) | Joint Simon task | | Exp1: (same-group Vs. different-group) AND (corresponding Vs. non-corresponding); Exp2: (cooperative Vs. competitive) AND (corresponding Vs. non-corresponding) | Exp1: 32 participants (22 women; age range 19–31 years); Exp2: 32 participants (18 women; age range 19–34 years) | | 2 | Reaction time | The activation of shared representations occurs only when individuals cooperate but not when they compete. | Significant condition (competitive or cooperate) × correspondence interaction (ηp2= .23). |
| (Jin et al., 2018) | Joint game with an adult followed by a gift selection task | | (Exp1: competitive and Exp2: cooperative ) (high coordination Vs. low coordination) | Exp1: 48 participants (high-coordination :Mage = 51 months 5 days,13 boys and 11 girls; low-coordination: Mage = 50 months 24 days,14 boys and 10 girls ); Exp2: (high-coordination: Mage = 53 months 12 days; 12 boys and 12 girls; low-coordination: Mage = 53 months 12 days, 12 boys and 12 girls) | | 2 | Perspective taking; Knowledge of other′s desire | Coordination enhanced children's performance in reasoning about diverse desires, allowing them to conduct desire inferences and perspective-taking. | Marginally significant effect of coordination on perspective taking (φ = 0.326). |
| (Le Bars et al., 2020) | Joint motor task: move a cursor and ultimately reach a target | | (Hight motor fluency Vs. low motor fluency) AND (balanced Vs. unbalanced motor role) AND (equal reward Vs. fair rewards Vs. all-or-none) | 35 participants (20 females; Mage = 23.64) | | 2 | Individual and joint feeling (judgement) of control | Individual JoC particularly relies on egocentric extrinsic cues while the collective JoC additionally relies on allocentric cues related to others' contributions. | Significant main effect of pivotality on individual JoC (p < 0.0001). Significant main effect of fluency on joint JoC (p < 0.0001); Significant main effect of pivotality on joint JoC (p < 0.0001), with an enhanced joint JoC for equal pivotality; Significant effect of rewards' context (i.e., equal, fair and random all-or-none) on joint JoC (p = 0.00022). |
| (Le Bars et al., 2022) | Moving a cursor and ultimately reach a target with motor noise and strategic noise | | (motor noise Vs. strategic noise) AND  (cooperative Vs. competitive) AND (equal leadership Vs. leader-follower) | Exp1: 40 participants (22 female; Mage = 26.92 years); Exp2: 60 participants ( 48 female; Mage = 22.84 years) | | 2 | Judgement of control (JOC); prosociality index; Team’s mean success and Team’s mean gain | The motor level of intentions significantly influences self-agency judgments, while the proximal levels of intentions, operationalized through the strategic-noise factor, appear to exert a greater impact on joint agency; Symmetrical role leads to more prosocial behavior. | Significant effect of motor noise on the individual judgement of control (p < .0001); significant effect of strategic noise on collective JOCs (p = .00035); significant effect of strategic noise on collective judgement of agency in dominant leader condition (p < .0001); Significant enhancement of collective JOCs in the case of prosocial strategies (p = .0438); Significant effect of leadership configuration on intentional prosocial behaviors (p = .018). |
| (Loehr & Vesper, 2016) | Musical transfer-of-learning (melody generation in coordination) | | Exp1: (individual goal Vs. shared goal) AND (alone Vs. together)  Exp2: (individual goal produced alone Vs. shared goal produced alone) | Exp1a: 32 participants (16 male, Mage=24.75); Exp1b: 32 participants (13 male; Mage = 21.47);Exp2: 32 participants (7 male; Mage = 22.56) | | 2 | Mean error rate (the number of incorrect trials divided by the total number of trials); | People engaged in a joint action make a representation of shared goals. | Significant effect of elicited goal (individual Vs. shared) on performance (Exp 1a: ηp2 = 0.19, Exp 1b: ηp2= 0.21). |
| (Loehr, 2018) | Sequence tone production task | | Explicit-cue Vs. Implicit-cue | Exp1: 100 participants (78 female, 21 male, 1 declined to indicate gender; mean age = 20.46); Exp2: 50 participants (34 female, 16 male; mean age = 20.54)  Exp3: 50 participants (37 female, 13 male; Mage = 20.04) | | 2 | Estimated mean control, estimated mean responsibility | People derive feelings of joint agency based on the success of the group as a whole; successful joint task performance leads to higher sense of joint agency. | Significant effect of error on joint agency in explicit-cue condition (Exp1: b=.40, p=0.001, Exp2: b=.35, p<0.001) and implicit-cue (Exp1: b=.13, p=0.021, Exp3: b=.19, p<0.001). |
| (McEllin & Michael, 2022) | Move a cursor at the edge of the screen to one of two targets in the middle of the screen  Followed by a donation game  (Exp1) or trust game (Exp2, 3, and 4) | | Signal Type (no-signal, redundant-signal, and useful-signal); Exp4: (no-signal, useful-signal) Vs. (intentional-signal, unintentional-signal) | Exp1: 72 participants (43 women, 29 men, Mage=35.41) ; Exp2: 72 participants (39 women, 33 men, Mage =34.69) ; Exp3: 72 participants (34 women and 38 men, Mage = 34.49) ; Exp4: 64 participants (28 women, 36 men, Mage = 36.15) | | 2 | Mean donation; mean investment | sensorimotor communication makes participants more generous to their partner, regardless of the utility of the signal; The effort that a co-actor invests in order to produce informative movement adaptations increases a participant's trust towards that co-actor. | Significant effect of signal type on donation (Exp1: ηp2 = .33) and investment behavior (Exp2: ηp2 = .11, Exp3: ηp2 = .31); Significant interaction between Trial type and signal type (Exp4: ηp2 = .09). |
| (McEllin et al., 2023) | Action coordination (coordinated drum taps) (Exp1) or decision-making coordination (joint object matching task) (Exp2) followed by a battery charging (persistence) task | | (Able-to-adapt condition Vs. unable-to-adapt) (belief about partner) AND (adaptive partner Vs. unadaptive partner) | Exp1: 28 participants (able-to-adapt condition) (15 females Mage = 23.4) and 28 participants (unable-to-adapt) (11 females ,Mage = 24.5); Exp2: 26 participants (able-to-adapt condition) (female = 15, Mage = 22.4) and 26 participants (unable-to-adapt) (female = 14, Mage = 21.3) | | 2 | Mean number of presses (commitment), Mean asynchrony ( timing similarity), Mean choice similarity | An agent's investment of effort to adapt movements or decisions to ensure successful and smooth coordination fosters a sense of commitment towards that agent. | Significant effect of partner (adaptive and unadaptive) on commitment (Exp1: η2 = .34 (after controlling for similarity η2 = .46), Exp2: η2 = .172 (after controlling for similarity η2 = .48)). |
| (Michael et al., 2016) | Action observation (participants watching videos of joint action) | | high coordination Vs. low coordination | Exp1a: 219 participants (127 females, Mage = 47.62); Exp1b: 207 participants (106 females, Mage = 46.97); Exp2: 376 participants (200 females, Mage = 47.81); Exp3: 209 participants (112 females, Mage = 47.25) | 2 (in the video) | | Perceived commitment (and multiple other measures) | Higher degree of coordination enhances the sense of commitment. | Significant Effect of coordination on perceived commitment (Exp1a: ηp2 = 0.019, Exp2:ηp2 = 0.014, Exp3: d = 0.340). |
| (Mitkidis et al., 2015) | LEGO building followed by a public goods game | | trust condition (public goods game) Vs. Control condition (no public goods game) | Lego game only: 74 participants (Mage = 23.5); Lego and the public goods game: 40 participants (average age: 23.3) | | 2 | Heart rate and heart rate synchrony, expectations of returns | An agent performs a task which involves the risk of trust only if they expected that their effort would be reciprocated. | Heart rate synchrony is positively associated with expectations of returns (p = .047). |
| (Obhi & Hall, 2011) | Tone production | | (Operant Vs. baseline) AND (Exp1: co-intention, Exp2: assigned intention) | 16 participants (10 women, 6 men, age range = 18–24) | | 2 | Intentional binding (pre-reflective sense of agency); perceived causal responsibility (subjective sense of self-agency) | Both initiator and responder develop intentional binding (pre-reflective sense of agency) and we-identity. | Significant main effect of condition (operant Vs. Baseline) on intentional binding (Exp1: p<.001, Exp2: p=.008) (shorter intervals under operant condition reflecting that intentional binding occurred). |
| (Ruys & Aarts, 2010) | Joint Simon task | | (Interdependency : independent Vs. cooperation Vs. competition) AND (response tone: high Vs. low) | Exp1:99 participants (undergraduates); Exp2: 59 participants (undergraduates) | | 2 | Response time; Simon effect | The activation of shared action representations not only occurs when people cooperate, but also when they compete. | Significant effect of interdependency on compatibility (cooperative versus individual Exp1: ηp2 = .06, competitive versus individual Exp1: ηp2 = .09). |
| (Saby et al., 2014) | Joint Simon task | | (Spatially compatible Vs. spatially incompatible) AND (collaborative Vs. competitive) | 62 participants (31 girls, Mage= 59.3 months) | | 2 (one adult experimenter) | Reaction time | Children have significantly lower response time when spatially compatible (stimulus in the same side as the key) than incompatible. | Significant effect of compatibility on mean reaction time (d = .274). |
| (Scharoun et al., 2017) | Pegboard task whereby a participant-confederate pair worked together to move a peg from one side of the board to the other | | helpful confederate Vs. less helpful confederate | 40 right-handed participants (Mage = 22.70) | | 2 | distance-confederate (average distance the confederate moved the peg from working space to the target end location) | Peg was moved farther in the helpful condition, resulting in a shorter distance left for the confederate to move the peg from working space to the target end location. | A significant effect of condition on the average distance-confederate (ƞ2 = .215). |
| (Shiraishi & Shimada, 2021) | Sequence tone production task | | (Alternation Vs. sequential AND (leader Vs. follower) | 36 right-handed male participants (Mage= 21.6) | | 2 | Mean agency rating; Mean behavioral coordination; EGG readings | Performing in alternation leads to higher sense of joint agency; coordination is more successful in alternating condition. | Significant effect of the task (alternation or sequential) on the sense of agency (p < 0.001); Significant effect of task on behavioral coordination (p< 0.005). |
| (Surtees et al., 2016) | Interactive perspective-taking game (judge the magnitude of a number either sat alone, or opposite a partner) | | (Exp1: (Alone Vs. Joint); Exp2: (alone Vs. joint first Vs. joint second); Exp3: (alone Vs. joint magnitude Vs. joint feature))  AND (consistent Vs. inconsistent) | Exp1: 32 participants ( 21 females, Mage = 21.7); Exp2: 48 participants (35 females, Mage = 20.81); Exp3: 48 participants (28 females, Mage = 22.5). | | 2 | Response time; error proportion | The mere presence of another person is not enough to evoke spontaneous processing of the partner's perspective. The simultaneous involvement of the partner is not necessary to trigger spontaneous perspective-taking as long as the partner has played an active role before. | Significant effect of consistency in joint condition (Exp1: p = 0.018, Exp2 (significant only in joint second): p = .001). significant effect of Consistency in the Joint-Magnitude Condition (Exp3: p = .012). |
| (Székely & Michael, 2018) | 2-player version of the classic snake game | | High effort VS low effort (effort invested by the other partner); Exp1: real partner, Exp2: partner is an algorithm, Exp3: own effort invested | Exp1: 26 participants (19 females, Mage = 23.04); Exp2:26 participants (18 females; Mage = 23.81); Exp3: 26 participants (18 females; Mage = 23.41) | | 2 | Persistence | participants' persisted longer at an increasingly boring joint action when they perceived what they believed to be cues of their partner's effortful contribution. | Effect of effort on commitment (Exp1: d = 0.475 (significant), Exp2: d = 0.102 (not significant), Exp3: d = 0.108 (not significant)). |
| (Török et al., 2019) | Touch-screen-based, sequential object-transfer task (move objects from one location to another) | | congruent trials incongruent trials, neutral trials | Exp1: 24 participants (7 male, Mage = 25.1); Exp2: 24 participants (12 male; Mage = 25.4) | | 2 | Proportion of efficient choices (action efficiency) | Actors made more coefficient decisions when co-efficiency entailed helping their partner by choosing the gap that was farther away (incongruent trials). | Main effect of condition on path choice (η2 = .43). |
| (Van der Weiden et al., 2019) | Joint Simon (go/no-go) task | | (Spatial compatibility: incompatible Vs. compatible) AND (action type: go Vs. nogo) × (experienced control question on no-go trials: action inhibition Vs. other's action) | 27 participants (undergraduate students) | | 2 (one virtual interaction partner) | Experience of control | Experienced control is higher for self-produced versus other-produced actions. | Significant effect of action type on experienced control (ηp2= 0.83). |
| (Van der Weiden et al., 2023) | An induction phase followed by a Joint Simon task. | | Exp1: (Low Agency Vs. High Agency) AND (Compatible Vs. Incompatible trials); Exp2: (unpredictable Vs. predictable) AND (incompatible Vs. compatible) | Exp1: 48 right-handed participants (15 males; Mage=23.9); Exp2: 85 participants (24 male; Mage=22.5) | | 2 | Reaction time | Self–other integration was weaker for the unpredictable (vs. predictable) co-actors. | Participants' compatibility effect was smaller when interacting with an unpredictable co-actor (general ɳ2 = 0.13) versus a predictable co-actor (general ɳ2 = 0.31). |
| (Van der Wel et al., 2012) | Rotate a pole by pulling on cords attached to the base of the pole on each side | | Individual-Individual (II) group; (Individual-Joint (IJ) group; Joint-Individual (JI) group; Joint-Joint (JJ) group | 105 participants (35 males and 70 females between the ages of 18 and 48) | | 2 | Combined error score (performance); Mean agency rating | Moving from joint to Individual actions increases the sense of self-agency; sense of agency for individually performed actions may be rooted in and shaped by prior learning experiences involving joint action. | Significant Effect of group type on the sense of self-agency (ηp2= .22). |
| (Wan & Zhu, 2021) | Joint music-making followed by block-Assembly Task and a dictator game (envelop task) | | fine-grained vs coarse-grained | 138 children (72 girls, Mage = 5 years and 6 months) | | 2 | Prosocial sharing (sharing blocks); number of donated stickers | Fine-grained coordination is more likely to enhance prosocial behaviors than coarse-grained coordination. | significant effects of coordination on cooperative behavior (block assembly task: β = 2.16, p = .010, OR = .12, envelop task: β = 1.56, p = .001, OR = .21). |
| (Wan et al., 2019) | Block arrangement followed by a star-arrangement and a dictator game (envelop task) | | coordination Vs. shared-goal-only | 129 participants (69 boys and 60 girls, Mage = 4 years and 6 months) | | 2 | Number of donated stickers | Level of coordination affects prosociality above and beyond having a shared goal. | significant effect of coordination on willingness to share (star-arrangement task: β = 1.62, p = .005, (envelop task: β = 1.07, p = .003). |
| (Wan et al., 2023) | Music making with a virtual partner followed by an online referential communication task | | (synchrony Vs. asynchrony Vs. antiphase synchrony) AND (privileged Vs. shared) | Exp1: 55 children (mean age = 6.2 years, 32 girls); Exp2: 45 children (mean age = 5.6 years, 21 girls) | | 2(a virtual partner) | Proportion of looks to interest areas (mean target ratio) | Playing instruments asynchronously or in alternation, but not synchronously, increases perspective-taking in children. | Significant effect of ground (privileged or shared) in antiphase synchrony coordination (target ratio: β = 0.10, p = 0.01, target-set ratio: β = 0.13, p<0.001); A similar ground effect for the asynchrony condition. |
| (Wolf et al., 2016) | Reaction time task (respond to a letter that briefly appeared on the screen by pressing a button on a mouse or response box) | | (joint attention Vs. disjoint attention) AND two (shared goal vs. individual goal) | 63 participants (20 males, Mage = 23.14) | | 2 | Social bonding; Response times; Attractiveness; Competitiveness | Joint attention can be sufficient to enhance the sense of social bonding. | The estimated mean bonding score of participants was between .6 and 6 points (scale 0 to 100) higher when rating their joint attention task partner (M = 49.5, SD = 12.4) compared with their disjoint attention task partner (M = 46.2, SD = 11.0). |
